# Immunotherapy for Digestive System Cancers: Progress, Challenges, and Future Directions

**DOI:** 10.3390/biomedicines14091919

**Published:** 2026-08-27

**Authors:** Keran Sun, Hongru Li, Hao Chi, Yuxuan Song, Yunze Niu, Jingyuan Ning, Hengrui Liu

**Affiliations:** 1 Key Laboratory of Immune Mechanism and Intervention on Serious Disease in Hebei Province, Department of Immunology, Hebei Medical University, Shijiazhuang 050017, China; sunkeran@stu.hebmu.edu.cn (K.S.); hongruli@stu.hebmu.edu.cn (H.L.); 2 Department of Gastroenterology, Fourth Hospital of Hebei Medical University, Shijiazhuang 050011, China; 3 Hebei Medical University Clinical Medicine Postdoctoral Research Station, Hebei Medical University, Shijiazhuang 050017, China; 4 John A. Burns School of Medicine, University of Hawai’i at Mānoa, Honolulu, HI 96813, USA; 5 Department of Urology, Peking University People’s Hospital, Beijing 100044, China; 6 Beijing Chaoyang Hospital, Capital Medical University, Beijing 100020, China; 7 Department of Biochemistry, University of Cambridge, Cambridge CB2 1QW, UK

**Keywords:** digestive system cancers, immunotherapy, immune checkpoint inhibitors, PD-1/PD-L1, MSI/dMMR, biomarkers, immune-related adverse events, multi-omics

## Abstract

Immune checkpoint blockade has changed the management of several digestive system cancers, but its impact is highly context dependent. This review evaluates evidence for esophageal, gastric and gastroesophageal junction, colorectal, hepatocellular, biliary tract and gallbladder, and pancreatic cancers. Randomized phase III trials have established chemoimmunotherapy or dual-checkpoint strategies in advanced esophageal cancer, biomarker- and regimen-dependent first-line therapy in gastric cancer, PD-1-based therapy for MSI-H/dMMR colorectal cancer, atezolizumab–bevacizumab and STRIDE for unresectable hepatocellular carcinoma, and chemoimmunotherapy for advanced biliary tract cancer. Recent results also expand perioperative treatment: neoadjuvant checkpoint blockade produces high pathological response rates in dMMR colon cancer, adjuvant atezolizumab plus mFOLFOX6 improves disease-free survival in stage III dMMR colon cancer, and perioperative serplulimab improves event-free survival in PD-L1-positive resectable gastric cancer. These advances coexist with important negative findings. Pembrolizumab-containing therapy did not meet superiority end points in KEYNOTE-062, the initial adjuvant signal in IMbrave050 was not sustained, and unselected pancreatic ductal adenocarcinoma remains largely resistant to checkpoint blockade. Early vaccine, cellular, TIGIT, radiomics, spatial, and multi-omics studies remain hypothesis-generating and require external or randomized validation. Clinical interpretation should integrate evidence maturity, biomarker validity, immune-related toxicity, patient-reported outcomes, cost, access, and manufacturing demands rather than response rate alone.

## 1. Introduction

Digestive system malignancies account for a substantial share of global cancer incidence and mortality. Global Cancer Statistics 2024 estimated that colorectal cancer represented 9.9% of new cancers worldwide, while stomach and liver cancers remained major causes of cancer death. These site-level statistics also show marked geographic heterogeneity, particularly for liver and gallbladder cancers, and caution against treating the digestive tract as one epidemiological entity. This review, therefore, focuses on the following six clinically distinct groups: esophageal cancer, gastric and gastroesophageal junction cancer, colorectal cancer, hepatocellular carcinoma, biliary tract and gallbladder cancer, and pancreatic ductal adenocarcinoma [1].

**Figure 1:**
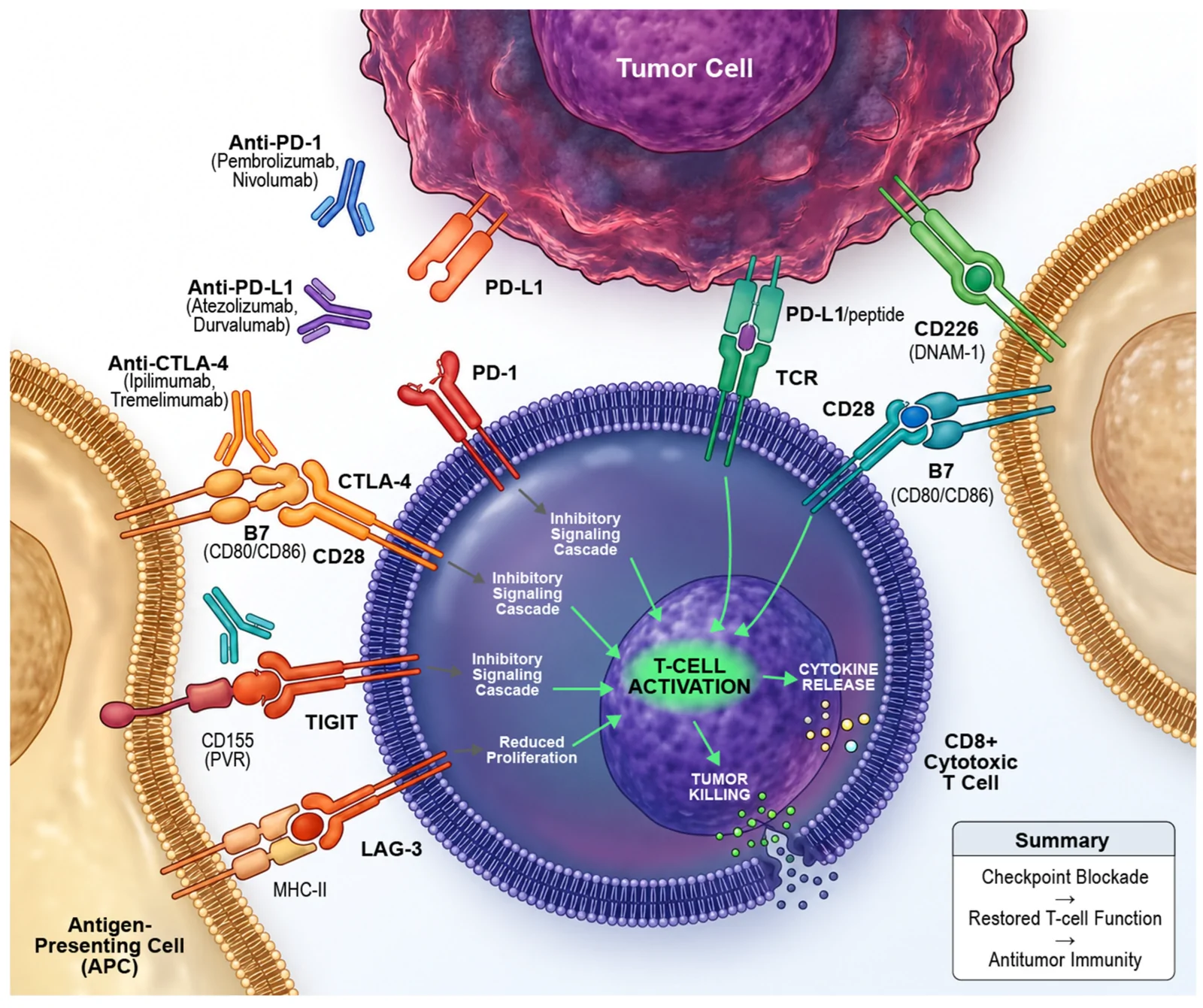
Immune checkpoint pathways and therapeutic blockade. PD-1–PD-L1 interactions can occur between tumor or antigen-presenting cells and activated T cells, whereas CTLA-4 competes with CD28 for B7 ligands primarily during T-cell priming. TIGIT binds CD155/CD112, and LAG-3 recognizes MHC class II and other ligands. Blocking these nonidentical inhibitory pathways can restore antitumor effector function but can also break peripheral tolerance and cause immune-related adverse events. The diagram is conceptual and not drawn to scale. Abbreviations: APC, antigen-presenting cell; CTLA-4, cytotoxic T-lymphocyte-associated protein 4; LAG-3, lymphocyte-activation gene 3; MHC, major histocompatibility complex; PD-1, programmed cell death protein 1; PD-L1, programmed death-ligand 1; TIGIT, T-cell immunoreceptor with immunoglobulin and ITIM domains.

Immune checkpoint inhibitors restore antitumor immunity by interrupting inhibitory pathways such as PD-1/PD-L1 and CTLA-4 (Figure 1), but response depends on tumor antigenicity, immune-cell composition, spatial organization, stromal exclusion, and treatment context. The contrast is clearest between mismatch repair-deficient or microsatellite instability-high tumors, which can be highly sensitive to PD-1 blockade, and most microsatellite-stable colorectal and pancreatic cancers, in which low immunogenicity and immune exclusion limit activity [2,3,4].

Immunotherapy refers mainly to immune checkpoint blockade, cancer vaccines, cellular therapy, and immune-modulating combinations. Zolbetuximab, a CLDN18.2-directed antibody with immune-effector functions, is also discussed, but it is considered separately from checkpoint inhibitors wherever the two strategies differ. Our emphasis is on practice-changing evidence and clinically relevant negative trials, together with emerging biomarker, perioperative, and translational strategies that may shape the next cycle of trials.

## 2. Literature Search and Review Scope

PubMed was searched through 4 August 2026 using combinations of disease terms for esophageal, gastric or gastroesophageal junction, colorectal, hepatocellular, biliary tract or gallbladder, and pancreatic cancers with intervention terms covering immune checkpoint inhibitors, PD-1, PD-L1, CTLA-4, TIGIT, cancer vaccines, CAR-T cells, perioperative therapy, biomarkers, adverse events, artificial intelligence, radiomics, spatial profiling, and multi-omics. The full PubMed strategy and the date of the final update are provided in Supplementary Table S1. Reference lists of pivotal trials and current clinical practice guidelines were checked to identify primary reports and mature follow-up analyses.

Evidence was selected for its ability to define current practice, alter interpretation of a major trial, or illustrate a clinically relevant limitation. Priority was given to peer-reviewed randomized trials, prospective clinical studies, mature follow-up reports, original translational analyses, patient-reported outcome studies, and authoritative guidelines. Meeting abstracts were used only when no peer-reviewed full report was available and are explicitly labeled. Case reports and very small case series were not used to support treatment recommendations. Preclinical studies were included only to explain mechanisms or to describe strategies that remain preclinical. Because the objective was comparative interpretation rather than exhaustive study identification or quantitative synthesis, no exhaustive study-selection flow diagram, pooled effect estimate, formal risk-of-bias instrument, or claim of comprehensive study inclusion is presented.

## 3. Esophageal Cancer

### 3.1. First-Line Treatment for Advanced Disease

KEYNOTE-590 established pembrolizumab plus platinum-fluoropyrimidine chemotherapy as a first-line option for advanced esophageal and gastroesophageal junction cancer (Table 1). In the peer-reviewed five-year update, 749 patients had been randomized. At a median follow-up of 58.8 months, median overall survival in the intention-to-treat population was 12.3 months with pembrolizumab plus chemotherapy and 9.8 months with placebo plus chemotherapy (HR 0.72, 95% CI 0.62–0.84); five-year overall survival rates were 10.6% and 3.0%, respectively. In the original analysis, median overall survival was 13.5 versus 9.4 months in the overall PD-L1 CPS at least 10 population and 13.9 versus 8.8 months in the ESCC subgroup with CPS at least 10, two populations that should not be conflated [5,6].

CheckMate-648 addressed a different population and used tumor-cell PD-L1 rather than CPS. The global phase III trial randomized 970 patients with previously untreated advanced esophageal squamous-cell carcinoma to nivolumab plus chemotherapy, nivolumab plus ipilimumab, or chemotherapy. In patients with tumor-cell PD-L1 expression of at least 1%, median overall survival was 15.4 versus 9.1 months with nivolumab plus chemotherapy (HR 0.54) and 13.7 versus 9.1 months with nivolumab plus ipilimumab (HR 0.64). Both regimens also improved overall survival in the overall population. Progression-free survival, however, was significantly improved only by nivolumab plus chemotherapy, not by the chemotherapy-free dual-checkpoint regimen. This distinction is relevant when balancing chemotherapy avoidance against early disease control and immune-related toxicity [7].

RATIONALE-306 should be considered separately rather than used as support for CheckMate-648. In 649 patients with advanced or unresectable esophageal squamous-cell carcinoma, tislelizumab plus chemotherapy prolonged median overall survival to 17.2 months versus 10.6 months with placebo plus chemotherapy (HR 0.66, 95% CI 0.54–0.80). Together, these trials support multiple first-line checkpoint-based regimens, but their PD-L1 assays, chemotherapy backbones, populations, and hierarchical testing strategies are not interchangeable [8].

### 3.2. Perioperative Therapy

Perioperative checkpoint blockade has produced high pathological response rates, although survival maturity varies. Keystone-001 was a prospective, single-arm phase II study in which 47 patients received neoadjuvant pembrolizumab plus chemotherapy and 46 were evaluable for efficacy. Major pathological response and pathological complete response occurred in 72% and 41%, respectively; two-year overall and disease-free survival estimates were encouraging but rely on a nonrandomized cohort and historical comparisons [9].

ESCORT-NEO provided randomized phase III evidence that adding camrelizumab can improve pathological response. Among 391 patients, pathological complete response rates were 28.0% with camrelizumab plus nab-paclitaxel/cisplatin, 15.4% with camrelizumab plus paclitaxel/cisplatin, and 4.7% with chemotherapy alone. Event-free survival, a co-primary end point, was not mature at publication. The appropriate conclusion is, therefore, that chemoimmunotherapy improved pathological response, not that it had already established a survival advantage [10].

### 3.3. Biomarkers and Emerging Checkpoints

PD-L1 remains the most clinically implemented biomarker in esophageal cancer, but assay type, scoring method, cutoff, histology, and regimen materially affect interpretation. TMB and MSI-H can identify highly immunogenic tumors across cancer types, yet disease-specific prospective validation is limited and MSI-H is uncommon in esophageal cancer. These considerations favor reporting the exact assay and threshold used in each trial rather than treating PD-L1 positivity as a universal binary category [11,12].

The TIGIT blockade illustrates the gap between biological rationale and clinical validation. In the interim phase Ib/II MORPHEUS-EC analysis, the cohorts were unequal, as follows: chemotherapy, n = 24; atezolizumab plus chemotherapy, n = 65; and tiragolumab plus atezolizumab plus chemotherapy, n = 63. The three-drug regimen yielded a higher objective response rate than atezolizumab plus chemotherapy (67.7% vs. 53.8%) and a progression-free survival HR of 0.66 (95% CI 0.44–0.99). Median overall survival was 16.0 versus 13.1 months, but the HR was 0.80 (95% CI 0.49–1.30). These small, interim data are hypothesis-generating and do not establish incremental survival benefit [13].

**Table 1:** Selected immunotherapy trials in esophageal cancer.

Trial	Design and Population	Experimental Treatment	Comparator	Key Result	Evidence Boundary	Source
KEYNOTE-590	Phase III; n = 749; untreated advanced esophageal/GEJ cancer	Pembrolizumab plus chemotherapy	Placebo plus chemotherapy	ITT OS 12.3 vs. 9.8 months; HR 0.72; 5-year OS 10.6% vs. 3.0%	Peer-reviewed 5-year update	[5,6]
CheckMate-648	Phase III; n = 970; untreated advanced ESCC	Nivolumab plus chemotherapy or nivolumab plus ipilimumab	Chemotherapy	PD-L1 at least 1% OS: 15.4 and 13.7 vs. 9.1 months; PFS significant only for nivolumab plus chemotherapy	Global main trial; tumor-cell PD-L1 assay	[7]
RATIONALE-306	Phase III; n = 649; advanced ESCC	Tislelizumab plus chemotherapy	Placebo plus chemotherapy	OS 17.2 vs. 10.6 months; HR 0.66	Independent tislelizumab trial	[8]
ESCORT-NEO	Phase III; n = 391; resectable ESCC	Two camrelizumab–chemo regimens	Chemotherapy	pCR 28.0%, 15.4%, and 4.7%	EFS immature at publication	[10]
Keystone-001	Single-arm phase II; n = 47 treated, n = 46 efficacy evaluable	Pembrolizumab plus chemotherapy	None	MPR 72%; pCR 41%	Historical comparison; no randomized survival inference	[9]
MORPHEUS-EC	Phase Ib/II interim analysis; unequal cohorts, n = 24/65/63	Tiragolumab plus atezolizumab plus chemotherapy	Atezolizumab plus chemotherapy and chemotherapy	ORR 67.7% vs. 53.8%; OS HR 0.80 (95% CI 0.49–1.30) vs. atezolizumab plus chemotherapy	Meeting abstract; hypothesis-generating	[13]

## 4. Gastric and Gastroesophageal Junction Cancer

### 4.1. First-Line Checkpoint Blockade

CheckMate 649 established nivolumab plus chemotherapy for HER2-negative advanced gastric, gastroesophageal junction, or esophageal adenocarcinoma, with the clearest evidence in PD-L1 CPS at least 5 (Table 2). At a minimum follow-up of 36.2 months, the overall survival HR was 0.70 (95% CI 0.61–0.81) in the CPS at least 5 population, and three-year overall survival was 21% versus 10%. A separate four-year Q-TWiST analysis addressed quality-adjusted time without symptoms or toxicity and should not be treated as an independent primary efficacy trial [14,15].

KEYNOTE-859 randomized 1579 patients and was positive in the intention-to-treat population: median overall survival was 12.9 versus 11.5 months (HR 0.78), median progression-free survival was 6.9 versus 5.6 months (HR 0.76), and objective response rates were 51.3% versus 42.0% with pembrolizumab plus chemotherapy and placebo plus chemotherapy, respectively. The magnitude of the benefit increased with PD-L1 expression. In the exploratory CPS below 1 subgroup, the overall survival HR was 0.929 (95% CI 0.732–1.177), so the results do not justify the statement that all patients benefit equally irrespective of PD-L1 [16].

KEYNOTE-062 provides an important negative and biomarker-qualified counterpoint. In the overall PD-L1 CPS at least 1 population, pembrolizumab monotherapy was noninferior but not superior to chemotherapy, and pembrolizumab plus chemotherapy did not meet the prespecified superiority end points. In the small exploratory MSI-H subgroup, median overall survival was not reached with pembrolizumab monotherapy versus 8.5 months with chemotherapy (HR 0.29, 95% CI 0.11–0.81), based on approximately 14 and 19 patients. The signal is biologically consistent with MSI-H sensitivity but is too small to establish a universal preference for monotherapy by itself [17].

**Table 2:** Selected immunotherapy and biomarker-directed trials in gastric and gastroesophageal junction cancer.

Trial	Design and Population	Experimental Treatment	Comparator	Key Result	Clinical Interpretation	Source
CheckMate 649	Phase III; n = 2031; HER2-negative advanced disease	Nivolumab plus chemotherapy	Chemotherapy	CPS at least 5 OS HR 0.70; 3-year OS 21% vs. 10%	Established first-line option; benefit enriched by PD-L1	[14]
KEYNOTE-859	Phase III; n = 1579; HER2-negative advanced disease	Pembrolizumab plus chemotherapy	Placebo plus chemotherapy	ITT OS 12.9 vs. 11.5 months; HR 0.78; ORR 51.3% vs. 42.0%	CPS below 1 HR 0.929; equal benefit across PD-L1 levels not established	[16]
KEYNOTE-062	Phase III; n = 763; CPS at least 1 advanced disease	Pembrolizumab with or without chemotherapy	Chemotherapy	Monotherapy noninferior but not superior overall; MSI-H exploratory OS HR 0.29	Small MSI-H subgroup; combination did not meet superiority	[17]
ASTRUM-006	Phase III; n = 588; resectable CPS at least 5 disease	Perioperative serplulimab plus SOX then serplulimab	Perioperative SOX	ITT EFS HR 0.73; CPS at least 10 EFS HR 0.65	OS immature; all randomized patients recruited in China	[18]
SPOTLIGHT	Phase III; n = 565; CLDN18.2-positive, HER2-negative advanced disease	Zolbetuximab plus mFOLFOX6	Placebo plus mFOLFOX6	PFS 10.61 vs. 8.67 months; OS 18.23 vs. 15.54 months; both HR 0.75	CLDN18.2-directed targeted antibody	[19]
GLOW	Phase III; n = 507; CLDN18.2-positive, HER2-negative advanced disease	Zolbetuximab plus CAPOX	Placebo plus CAPOX	PFS HR 0.687; OS HR 0.771	Independent validation of CLDN18.2 targeting	[20]

### 4.2. Perioperative Immunotherapy

Perioperative development is rapidly changing. In the 2026 ASTRUM-006 phase III trial, 588 patients with PD-L1 CPS at least 5 resectable gastric or gastroesophageal junction adenocarcinoma were randomized to neoadjuvant serplulimab plus SOX followed by adjuvant serplulimab or perioperative SOX. Event-free survival improved both in the CPS at least 10 population (HR 0.65, 95% CI 0.47–0.90) and in the intention-to-treat population (HR 0.73, 95% CI 0.56–0.94). Overall survival remains immature, and all randomized patients were recruited in China, so broader generalizability and the contribution of the chemotherapy-sparing adjuvant component require further follow-up [18].

### 4.3. CLDN18.2 and Molecular Stratification

Zolbetuximab is a CLDN18.2-directed antibody rather than an immune checkpoint inhibitor. In SPOTLIGHT, zolbetuximab plus mFOLFOX6 improved median progression-free survival to 10.61 versus 8.67 months and median overall survival to 18.23 versus 15.54 months, with HRs of 0.75 for both outcomes. In GLOW, zolbetuximab plus CAPOX improved median progression-free survival to 8.21 versus 6.80 months (HR 0.687) and median overall survival to 14.39 versus 12.16 months (HR 0.771). These trials establish CLDN18.2 as a treatment-selection biomarker for targeted antibody therapy, not as a validated predictor of checkpoint inhibition [19,20].

MSI-H, EBV positivity, TMB, stromal programs, and spatial immune organization may refine response prediction. Exploratory CheckMate 649 analyses associated hypermutation and, to a lesser extent, EBV-positive disease with greater relative benefit from nivolumab-containing treatment, while low stromal signatures also appeared favorable. Because only a subset of randomized patients had evaluable sequencing and the analyses were post hoc, these signatures require prospective validation and should not replace established MSI/MMR, HER2, PD-L1, or CLDN18.2 testing [21].

## 5. Colorectal Cancer

### 5.1. Metastatic MSI-H/dMMR Disease

**Figure 2:**
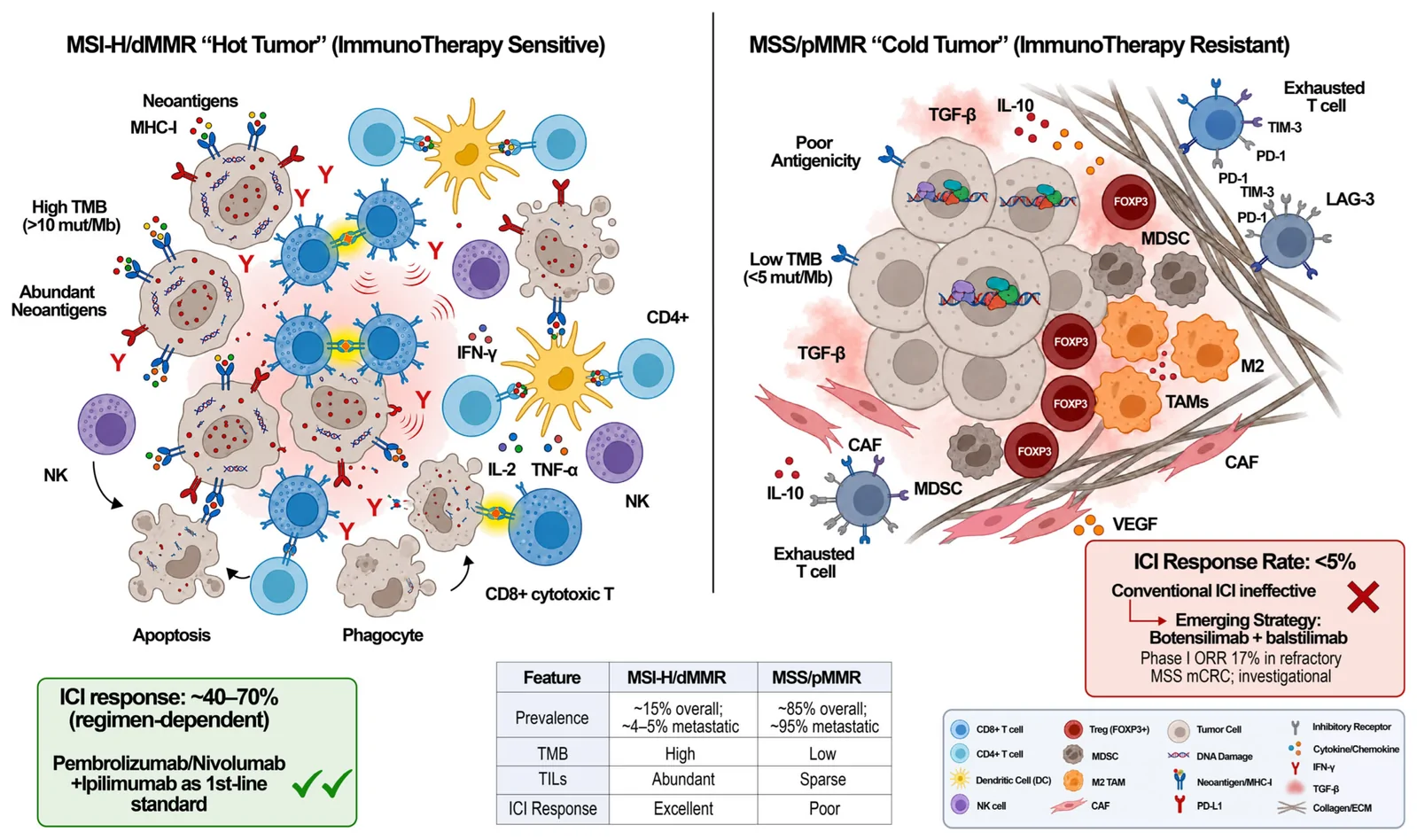
Immune contexture and treatment evidence in MSI-H/dMMR and MSS/pMMR colorectal cancer. MSI-H/dMMR tumors have higher neoantigen load and more frequent lymphocytic infiltration than most MSS/pMMR tumors. In metastatic disease, MSI-H/dMMR represents approximately 4–5% of cases and is associated with substantial activity from PD-1-based therapy; response varies by regimen and treatment line. Most MSS/pMMR tumors remain resistant because of low immunogenicity, stromal exclusion, suppressive myeloid cells, and TGF-β-associated programs. Botensilimab plus balstilimab achieved a 17% confirmed response rate in a phase I MSS metastatic colorectal cancer cohort and remains investigational. The figure separates validated treatment from exploratory strategies. Abbreviations: CAF, cancer-associated fibroblast; dMMR, deficient mismatch repair; ICI, immune checkpoint inhibitor; MDSC, myeloid-derived suppressor cell; MSI-H, microsatellite instability-high; MSS, microsatellite stable; pMMR, proficient mismatch repair; TMB, tumor mutational burden.

KEYNOTE-177 established first-line pembrolizumab in MSI-H/dMMR metastatic colorectal cancer (Figure 2 and Table 3). In the five-year update, 307 patients had been randomized and the effective crossover rate from chemotherapy to PD-1/PD-L1 blockade was 62%. Median overall survival was 77.5 months with pembrolizumab versus 36.7 months with chemotherapy (HR 0.73, 95% CI 0.53–0.99), median progression-free survival was 16.5 versus 8.2 months (HR 0.60, 95% CI 0.45–0.79), and five-year overall survival was 54.8% versus 44.2%. Grade 3–5 adverse events were less frequent with pembrolizumab than with chemotherapy [3,22].

The original KEYNOTE-177 analysis reported progressive disease as the best RECIST response in 29.4% of pembrolizumab-treated patients. A separate retrospective cohort of 123 patients found pseudoprogression in 10% of the entire cohort and in 52% of early primary radiographic progressions, all within the first three months. These data do not show that half of KEYNOTE-177 progressions were pseudoprogression. They instead support repeat imaging under immune-response criteria only for clinically stable patients with early unconfirmed progression [23].

CheckMate 8HW evaluated two distinct randomized comparisons. In previously untreated, centrally confirmed MSI-H/dMMR metastatic colorectal cancer, nivolumab plus ipilimumab improved 24-month progression-free survival to 72% versus 14% with chemotherapy (HR 0.21, 97.91% CI 0.13–0.35). Across all treatment lines, nivolumab plus ipilimumab improved progression-free survival versus nivolumab monotherapy (median not reached vs. 39.3 months; HR 0.62, 95% CI 0.48–0.81) and increased objective response from 58% to 71%; complete response rates were 28% and 30%, respectively. The two comparisons require separate interpretation because the controls and analysis populations differ [24,25].

### 5.2. Localized dMMR Colon and Rectal Cancer

Neoadjuvant treatment has produced exceptionally high pathological response rates in localized dMMR colon cancer. NICHE-2 enrolled 115 patients, 111 of whom were included in the efficacy analysis. Pathological response occurred in 98%, including major pathological response in 95% and pathological complete response in 68%. At the peer-reviewed publication cutoff, no recurrence had occurred after a median follow-up of 26 months. A subsequent ESMO 2024 late-breaking update reported 100% three-year disease-free survival after a median postoperative follow-up of 36.5 months; this is a meeting update and should not be attributed to the original paper [26,27].

NICHE-3 extended neoadjuvant investigation to nivolumab plus relatlimab. In this single-arm phase II study, 57 of 59 patients had a pathological response (97%), 54 had a major pathological response (92%), and 40 had a pathological complete response (68%). These results support further evaluation but do not establish superiority over the short-course nivolumab–ipilimumab strategy [28].

For locally advanced dMMR rectal cancer, a small prospective study showed that dostarlimab could produce sustained clinical complete responses and allow for nonoperative management during the reported follow-up. This organ-preservation strategy is promising, but long-term local control, salvageability, functional outcomes, and applicability outside highly selected centers remain central questions [29].

The 2026 ATOMIC trial moved immunotherapy into randomized adjuvant treatment. Among 712 patients with resected stage III dMMR colon cancer, atezolizumab plus mFOLFOX6 improved three-year disease-free survival to 86.3% versus 76.2% with mFOLFOX6 alone (HR 0.50, 95% CI 0.35–0.73). Grade 3 or 4 adverse events occurred in 84.1% and 71.9%, respectively. The result is practice relevant but should be interpreted alongside the added year of treatment and higher toxicity burden [30].

### 5.3. MSS/pMMR Disease and Immune Exclusion

Most metastatic colorectal cancers are MSS/pMMR and remain resistant to conventional checkpoint blockade. The phase III IMblaze370 trial found no overall survival advantage for atezolizumab plus cobimetinib or atezolizumab alone over regorafenib. The randomized BACCI study similarly showed that adding atezolizumab to capecitabine and bevacizumab did not establish a transformative benefit. These failures demonstrate that mechanistic plausibility and early activity do not substitute for randomized validation [31,32].

Botensilimab, an Fc-engineered anti-CTLA-4 antibody, has shown an early activity signal in refractory MSS disease but remains investigational. In the published phase I study, 148 patients received botensilimab plus balstilimab and 101 were response evaluable with at least six months of follow-up. The confirmed objective response rate was 17%, the disease control rate was 61%, and median progression-free survival was 3.5 months. In an exploratory analysis, the objective response was 22% among 77 patients without active liver metastases and 0% among 24 patients with active liver metastases. Disease burden, aggressive tumor biology, cohort enrichment, and selection bias prevent causal attribution to hepatic metastasis itself [33].

Early NEST data provide proof of concept for neoadjuvant botensilimab plus balstilimab in resectable pMMR/MSS colorectal cancer, but the evidence consists of very small meeting-abstract cohorts with changing treatment durations and evaluable denominators. These versions should not be merged into one sample size or response rate [34].

**Table 3:** Selected immunotherapy trials in colorectal cancer.

Trial	Setting and Design	Treatment	Comparator	Key Result	Evidence Boundary	Source
KEYNOTE-177	Phase III; n = 307; first-line MSI-H/dMMR mCRC	Pembrolizumab	Chemotherapy	PFS 16.5 vs. 8.2 months; HR 0.60; 5-year OS 54.8% vs. 44.2%	62% effective crossover; pseudoprogression data derive from another cohort	[3,22]
CheckMate 8HW: combination vs. chemotherapy	Phase III; n = 303 randomized; first-line MSI-H/dMMR mCRC	Nivolumab plus ipilimumab	Chemotherapy	24-month PFS 72% vs. 14%; HR 0.21	Centrally confirmed primary population n = 255	[24]
CheckMate 8HW: combination vs. nivolumab	Phase III; n = 707 randomized; all treatment lines	Nivolumab plus ipilimumab	Nivolumab	PFS HR 0.62; ORR 71% vs. 58%; CR 30% vs. 28%	Different comparator and population from chemotherapy analysis	[25]
NICHE-2	Single-arm phase II; n = 115 enrolled, n = 111 efficacy evaluable; localized dMMR colon cancer	Short-course nivolumab plus ipilimumab	None	Pathological response 98%; MPR 95%; pCR 68%; no recurrence at median 26 months	3-year DFS 100% derives from a meeting update	[26,27]
NICHE-3	Single-arm phase II; n = 59; localized dMMR colon cancer	Nivolumab plus relatlimab	None	Pathological response 97%; MPR 92%; pCR 68%	Promising nonrandomized evidence	[28]
ATOMIC	Phase III; n = 712; resected stage III dMMR colon cancer	Atezolizumab plus mFOLFOX6	mFOLFOX6	3-year DFS 86.3% vs. 76.2%; HR 0.50	Grade 3–4 AEs 84.1% vs. 71.9%	[30]
IMblaze370	Phase III; n = 363; previously treated, predominantly MSS mCRC	Atezolizumab with or without cobimetinib	Regorafenib	No OS improvement; combination HR 1.00	Randomized negative trial	[31]
Botensilimab plus balstilimab	Phase I; n = 148 treated, n = 101 response evaluable; refractory MSS mCRC	Fc-engineered CTLA-4 plus PD-1 blockade	None	ORR 17%; DCR 61%; PFS 3.5 months	Liver-metastasis subgroup exploratory and nonrandomized	[33]

## 6. Hepatocellular Carcinoma

### 6.1. Global Burden and First-Line Standards

In 2024, liver cancer, defined at the site level as ICD-10 C22 and, therefore, including intrahepatic bile duct cancer, accounted for an estimated 843,045 new cases and 732,489 deaths worldwide. These values should not be described as HCC-specific counts, although HCC constitutes the predominant primary liver cancer histology [1].

IMbrave150 established atezolizumab plus bevacizumab for systemic-treatment-naive unresectable HCC (Table 4). In 501 patients, the median overall survival was 19.2 versus 13.4 months with sorafenib (HR 0.66, 95% CI 0.52–0.85), and the median progression-free survival was 6.9 versus 4.3 months (HR 0.65, 95% CI 0.53–0.81). Grade 3–4 treatment-related adverse events occurred in 43% and 46%, respectively. The regimen requires attention to bleeding risk, varices, hypertension, proteinuria, and liver function in addition to immune-related toxicity [35,36].

HIMALAYA established the STRIDE regimen of a single priming dose of tremelimumab plus regular-interval durvalumab. Among 1171 randomized patients, STRIDE improved overall survival over sorafenib. At the five-year exploratory update, the overall survival HR was 0.76 (95% CI 0.65–0.89), and the 60-month survival was 19.6% versus 9.4%. No new late-onset treatment-related serious adverse-event signal was reported [37,38].

Not every rational combination succeeds. LEAP-002 did not cross its prespecified multiplicity-adjusted significance boundaries for overall or progression-free survival with lenvatinib plus pembrolizumab compared with lenvatinib alone. In the 2026 randomized phase II component of TRIPLET-HCC, adding low-dose ipilimumab to atezolizumab plus bevacizumab did not meet the response threshold required to proceed to phase III and increased treatment-related mortality. These studies caution against assuming that adding a third immune or targeted agent will improve net benefit [39,40].

### 6.2. Perioperative Immunotherapy

IMbrave050 initially reported a recurrence-free survival signal for adjuvant atezolizumab plus bevacizumab after high-risk resection or ablation. Longer follow-up changed the interpretation. At a median follow-up of 35.1 months, median recurrence-free survival was 33.2 versus 36.0 months (HR 0.90, 95% CI 0.72–1.12), and overall survival remained immature (HR 1.26, 95% CI 0.85–1.87). The updated benefit–risk profile does not support routine adjuvant use in an unselected high-risk population [41,42].

### 6.3. Biomarkers and Cellular Therapy

Exploratory translational analyses of atezolizumab–bevacizumab associated higher CD274 expression, T-effector signatures, and CD8 T-cell density with more favorable outcomes, whereas other stromal or oncofetal programs were associated with less benefit. No biomarker, including PD-L1, has been prospectively validated to select patients with HCC for first-line immunotherapy [43].

GPC3-directed CAR-T cells remain in an early phase. Two first-in-human phase I studies included only 13 patients with GPC3-positive advanced HCC and produced two partial responses. Cytokine-release syndrome occurred in 9 of 13 patients, including one grade 5 event. The findings indicate preliminary activity, not established efficacy or a definitive safety profile [44].

**Table 4:** Selected immunotherapy trials in hepatocellular carcinoma.

Trial	Design and Setting	Experimental Treatment	Comparator	Key Result	Safety or Limitation	Source
IMbrave150	Phase III; n = 501; first-line unresectable HCC	Atezolizumab plus bevacizumab	Sorafenib	OS 19.2 vs. 13.4 months; HR 0.66; PFS HR 0.65	Requires bleeding-risk and anti-VEGF assessment	[35,36]
HIMALAYA	Phase III; n = 1171; first-line unresectable HCC	STRIDE	Sorafenib	5-year OS 19.6% vs. 9.4%; HR 0.76	Long-term exploratory update; no new late serious toxicity signal	[37,38]
LEAP-002	Phase III; n = 794; first-line advanced HCC	Lenvatinib plus pembrolizumab	Lenvatinib	OS HR 0.84; PFS HR 0.87	Did not cross prespecified multiplicity-adjusted significance boundaries	[39]
TRIPLET-HCC	Randomized phase II; n = 226 treated	Atezolizumab, bevacizumab, and ipilimumab	Atezolizumab plus bevacizumab	24-week ORR 30% vs. 27%; phase III threshold not met	Treatment-related death 5% vs. 0%	[40]
IMbrave050 updated	Phase III; n = 668; adjuvant high-risk HCC	Atezolizumab plus bevacizumab	Active surveillance	Updated RFS HR 0.90; OS HR 1.26, immature	Initial RFS advantage not sustained	[42]
GPC3 CAR-T	Two phase I studies; n = 13; advanced GPC3-positive HCC	GPC3-directed CAR-T	None	Two partial responses	CRS in 9/13, including one grade 5 event	[44]

## 7. Biliary Tract and Gallbladder Cancer

### 7.1. Disease Scope and First-Line Chemoimmunotherapy

Gallbladder cancer should not be treated as synonymous with the broader BTC population (Table 5). Global Cancer Statistics 2024 estimated approximately 126,000 new gallbladder cancer cases and 92,000 deaths, with a substantially greater burden among women. By contrast, pivotal systemic-treatment trials pooled intrahepatic, extrahepatic, and gallbladder primary sites [1].

TOPAZ-1 established durvalumab plus gemcitabine and cisplatin as a first-line option for advanced BTC. In the latest four-year post hoc analysis, the median overall survival was 13.0 versus 11.4 months (HR 0.75, 95% CI 0.64–0.88), and the 48-month survival was 11.8% versus 4.3%. The primary trial enrolled 685 patients, including 171 with gallbladder cancer. In the primary analysis, the gallbladder subgroup overall survival HR was 0.94 (95% CI 0.65–1.37); the subgroup was not powered for a definitive site-specific comparison. The correct conclusion is, therefore, a BTC-level standard that included gallbladder cancer, not separately proven gallbladder-specific efficacy [45,46,47].

KEYNOTE-966, likewise, established pembrolizumab plus gemcitabine and cisplatin at the BTC level. Among 1069 patients, the median overall survival was 12.7 versus 10.9 months (HR 0.83, 95% CI 0.72–0.95). The gallbladder subgroup included 233 patients and had an overall survival HR of 0.96 (95% CI 0.73–1.26). Response rates were 29% in both randomized groups, although the response duration was longer with pembrolizumab. These results reinforce the need to distinguish the overall-trial benefit from uncertain anatomic-subgroup effects [48].

### 7.2. Perioperative and Later-Line Evidence

The randomized phase II ACCORD trial enrolled 93 patients with resected extrahepatic cholangiocarcinoma or gallbladder cancer, only 22 of whom had gallbladder cancer. Camrelizumab plus chemoradiotherapy improved overall and recurrence-free survival compared with observation, but the inactive comparator, small and imbalanced gallbladder subgroup, and multimodality intervention prevent attributing benefit specifically to camrelizumab or generalizing the pooled result to gallbladder cancer [49].

Dual-checkpoint blockade has generated a signal in small later-line cohorts. The SWOG S1609 cohort 48 included 19 previously treated patients with advanced gallbladder cancer; nivolumab plus ipilimumab produced a confirmed objective response rate of 16% and a median response duration of 14.8 months. A 2026 phase II MoST-CIRCUIT cohort enrolled 60 patients with intrahepatic cholangiocarcinoma or gallbladder cancer and reported an overall objective response rate of 12%, with exploratory rates of 3% and 26% in the two anatomic groups; severe immune-related adverse events occurred in 20%. These nonrandomized data do not establish a standard but support site-aware investigation [50,51].

### 7.3. PD-L1, Methylation, and Candidate Biomarkers

PD-L1 expression should not be presented as a validated BTC treatment-selection marker. TOPAZ-1 and KEYNOTE-966 did not show a clear, prospectively validated threshold that identified patients who did or did not benefit from adding immunotherapy. A 47-case gallbladder pathology series detected PD-L1 in 46 tumors (98%) and PD-1-positive tumor-infiltrating lymphocytes in 37 cases (78.7%); the original manuscript reversed these quantities. The small, retrospective, assay-specific study describes prevalence rather than predictive validity [52].

An integrative methylome study of 105 BTC tumors, including 48 gallbladder cancers, associated fewer common methylation changes with longer survival, an inflamed immune microenvironment, CD8 infiltration, and PD-L1 expression. Because response to checkpoint blockade was not evaluated, this signature is prognostic and hypothesis-generating rather than a validated predictive biomarker [53].

**Table 5:** Selected immunotherapy trials in biliary tract and gallbladder cancer.

Trial	Design and Population	Treatment	Comparator	Key Result	Gallbladder-Specific Boundary	Source
TOPAZ-1	Phase III; n = 685; untreated advanced BTC	Durvalumab plus gemcitabine/cisplatin	Placebo plus gemcitabine/cisplatin	Primary OS HR 0.80; 4-year post hoc OS HR 0.75	GBC, n = 171; primary subgroup OS HR 0.94 (95% CI 0.65–1.37)	[45,47]
KEYNOTE-966	Phase III; n = 1069; untreated advanced BTC	Pembrolizumab plus gemcitabine/cisplatin	Placebo plus gemcitabine/cisplatin	OS 12.7 vs. 10.9 months; HR 0.83	GBC, n = 233; subgroup OS HR 0.96 (95% CI 0.73–1.26)	[48]
ACCORD	Randomized phase II; n = 93; resected extrahepatic CCA or GBC	Camrelizumab plus chemoradiotherapy	Observation	OS HR 0.43; RFS HR 0.46	Only 22 GBC; inactive comparator; multimodality effect	[49]
SWOG S1609 cohort 48	Nonrandomized phase II; n = 19; pretreated advanced GBC	Nivolumab plus ipilimumab	None	Confirmed ORR 16%; median response duration 14.8 months	Signal only; one possibly treatment-related death	[50]
MoST-CIRCUIT	Single-arm phase II; n = 60; iCCA or GBC	Nivolumab plus ipilimumab	None	Overall ORR 12%; exploratory GBC ORR 26%	GBC, n = 23; severe irAEs 20% overall	[51]
Methylome study	Integrative cohort; n = 105 BTC plus TCGA-CHOL	Not an intervention	Not applicable	Low methylation-change phenotype associated with survival and inflamed TIME	No ICI-treated validation cohort; not predictive	[53]

## 8. Pancreatic Ductal Adenocarcinoma

### 8.1. Why Conventional Checkpoint Blockade Usually Fails

**Figure 3:**
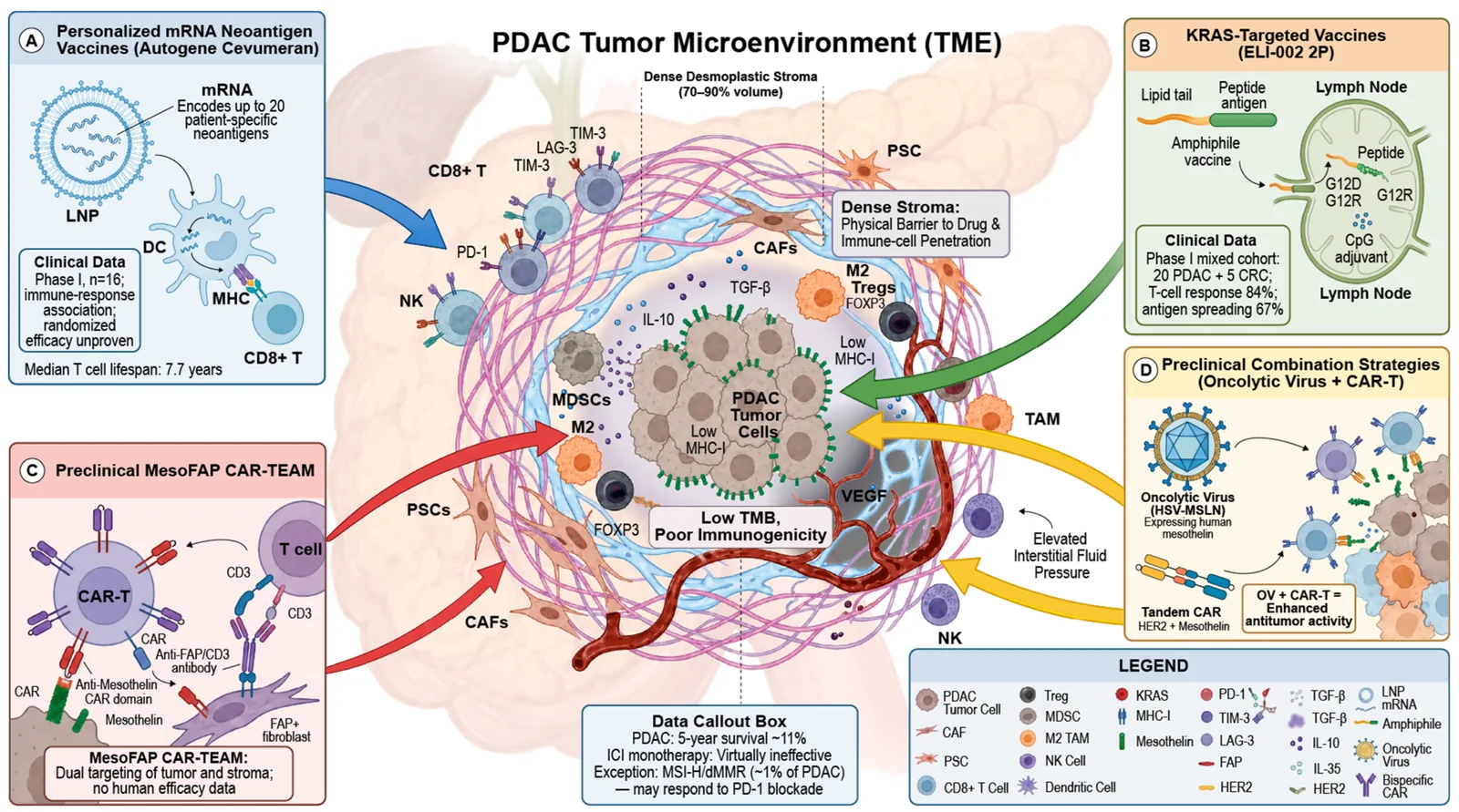
Pancreatic ductal adenocarcinoma immune exclusion and emerging strategies. Dense desmoplastic stroma, suppressive myeloid and fibroblast programs, low baseline immunogenicity, and limited effector T-cell access contribute to checkpoint resistance in most PDAC. PD-1 blockade is clinically relevant mainly for the rare MSI-H/dMMR subset. Autogene cevumeran and ELI-002 have shown immunogenicity in small, nonrandomized cohorts; outcome associations do not establish efficacy. Transient mesothelin CAR-T cells have produced stable disease in an early six-patient clinical study, while dual tumor–stroma CAR-T and oncolytic-virus combinations remain preclinical. GPC3 is not presented as a PDAC target. Abbreviations: CAF, cancer-associated fibroblast; CAR-T, chimeric antigen receptor T cell; dMMR, deficient mismatch repair; MDSC, myeloid-derived suppressor cell; MSI-H, microsatellite instability-high; PDAC, pancreatic ductal adenocarcinoma; TME, tumor microenvironment; VEGF, vascular endothelial growth factor.

PDAC is characterized by low baseline immunogenicity in most patients, dense desmoplastic stroma, suppressive myeloid and fibroblast programs, and limited effector T-cell access (Figure 3 and Table 6). These features help explain why responses to checkpoint monotherapy are rare outside the small MSI-H/dMMR subset. The relevant exception is PD-1 blockade, not a generic claim about PD-L1 inhibitors [2,54].

Randomized evidence remains negative for unselected disease. In PA.7, 180 patients with untreated metastatic PDAC were randomized to gemcitabine and nab-paclitaxel with or without durvalumab and tremelimumab. Median overall survival was 9.8 versus 8.8 months (*p* = 0.72), and the addition of dual-checkpoint blockade did not improve clinical outcomes. A 2026 phase I platform study reported encouraging response signals in two 15-patient chemoimmunotherapy cohorts, but the nonrandomized design, small samples, toxicity, and lack of expansion preclude a change in standard treatment [55,56].

### 8.2. Therapeutic Vaccines

Autogene cevumeran has demonstrated immunogenicity in a small adjuvant phase I study. Eight of sixteen vaccinated patients generated high-magnitude neoantigen-specific T-cell responses. At a median follow-up of 3.2 years, median recurrence-free survival was not reached in immune responders versus 13.4 months in nonresponders (*p* = 0.007), and vaccine-induced CD8 T-cell clones remained detectable. The 2025 report is an extended follow-up of the same 16-patient cohort, not independent validation. Because the outcome was compared by an on-treatment immune response in a nonrandomized sample, the study does not establish vaccine efficacy; randomized evaluation is ongoing [57,58].

AMPLIFY-201 evaluated ELI-002 2P in 25 patients with molecular residual disease, including 20 with PDAC and 5 with colorectal cancer. Mutant KRAS-specific T-cell responses occurred in 21 of 25 patients (84%), and antigen spreading occurred in 67% of evaluable patients. Stronger on-treatment responses were associated with longer radiographic recurrence-free and overall survival, but the single-arm, mixed-tumor design and response-defined analyses preclude causal claims of efficacy in PDAC [59,60].

### 8.3. Cellular and Microenvironment-Directed Approaches

Clinical evidence for CAR-T therapy in PDAC is limited. In a six-patient phase I study of transient mesothelin-directed CAR-T cells, no dose-limiting toxicity, cytokine-release syndrome, or neurotoxicity was observed, but the best RECIST outcome was stable disease. Dual tumor–stroma targeting with MesoFAP CAR-TEAM and oncolytic-virus-assisted mesothelin targeting have improved activity in patient-derived models and mice, but both remain preclinical. They should not be presented as established therapeutic options [61,62,63].

The distinction between immune activation and net clinical benefit is especially important in PDAC. Degrading stroma or adding checkpoint agents can alter immune markers without improving survival, and aggressive stromal depletion may have context-dependent effects. Future studies need prospectively defined biomarkers, pharmacodynamic evidence, randomized controls, and feasibility measures that include manufacturing time and treatment-window attrition.

**Table 6:** Selected immunotherapy evidence in pancreatic ductal adenocarcinoma.

Strategy	Study and Design	Population	Key Result	Evidence Level	Main Limitation	Source
Dual ICI plus chemotherapy	PA.7 randomized phase II; n = 180	Untreated metastatic PDAC	OS 9.8 vs. 8.8 months; *p* = 0.72	Randomized negative	No benefit in unselected disease	[55]
PD-1 blockade	Mismatch-repair-deficient tumor study	Rare MSI-H/dMMR PDAC subset	Responses observed across dMMR tumors	Biomarker-defined clinical evidence	Very low prevalence in PDAC	[2]
Autogene cevumeran	Adjuvant phase I; n = 16; extended follow-up of same cohort	Resected PDAC	8/16 immune responders; RFS association maintained at 3.2 years	Early nonrandomized	Response-defined comparison does not establish efficacy	[57,58]
ELI-002 2P	AMPLIFY-201 single-arm phase I; n = 25	20 PDAC and 5 CRC with molecular residual disease	KRAS-specific T-cell response 84%; antigen spreading 67%	Early mixed tumor	On-treatment response association; no control	[60]
Mesothelin CAR-T	Phase I; n = 6	Chemotherapy-refractory metastatic PDAC	Best RECIST outcome: stable disease	Early clinical feasibility	Very small sample; no objective response	[61]
MesoFAP CAR-TEAM	Preclinical	PDAC models	Dual tumor–stroma activity	Preclinical	No human efficacy or safety data	[62]
HSV-MSLN plus mesothelin CAR-T	Preclinical	Cell and mouse PDAC models	Enhanced antigen expression and antitumor activity	Preclinical	No human data	[63]

## 9. Comparative Biomarkers and Precision Stratification

Biomarkers in digestive cancer immunotherapy answer different questions and should not be placed in a single undifferentiated category. MSI-H/dMMR is both a biological subtype and a validated treatment-selection marker for PD-1-based therapy in colorectal cancer and several other advanced solid tumors. PD-L1 is a regimen- and assay-dependent enrichment marker in esophageal and gastric cancers, but it has not provided a validated selection threshold in HCC or BTC. TMB has tumor-agnostic regulatory relevance at specific thresholds, yet disease-specific prevalence, sequencing platforms, and overlap with MSI limit direct transfer across cancers [2,11].

CLDN18.2 is a validated treatment-selection biomarker for zolbetuximab in HER2-negative gastric and gastroesophageal junction adenocarcinoma, but it is not a checkpoint-response biomarker (Table 7). EBV positivity, T-effector signatures, spatial immune-cell arrangements, stromal programs, methylation phenotypes, and post-treatment immune responses are promising stratification variables, yet most remain exploratory. A clinically useful table must, therefore, specify whether a marker is predictive, prognostic, diagnostic, or a direct treatment target and must state the assay, threshold, disease, treatment, and validation level.

**Table 7:** Comparative biomarker framework across digestive system cancers.

Biomarker	Cancer and Treatment Context	Clinical Role	Assay or Threshold	Evidence Maturity	Key Limitation	Source
MSI-H/dMMR	CRC; gastric; tumor-agnostic advanced disease	Validated predictor of PD-1-based therapy	IHC, PCR, or validated NGS	High in metastatic CRC; expanding perioperative evidence	Low prevalence in PDAC, esophageal cancer, and most BTC	[2,3]
PD-L1	Esophageal and gastric checkpoint regimens	Regimen-specific enrichment marker	CPS, TPS, or tumor-cell score as prespecified	Prospective phase III but assay dependent	Thresholds and scoring systems are not interchangeable	[7,16]
PD-L1	HCC and BTC/GBC	Exploratory association	Assay specific	Not validated for treatment selection	Large trials do not establish a clinical threshold	[43,48]
TMB-high	Tumor-agnostic pembrolizumab context	Regulatory and enrichment biomarker	At least 10 mutations/Mb in the qualifying assay	Prospective basket evidence	Platform, tumor type, and overlap with MSI matter	[11]
CLDN18.2	HER2-negative gastric/GEJ adenocarcinoma	Direct treatment-selection target for zolbetuximab	Trial-validated IHC threshold	Two randomized phase III trials	Not a checkpoint-response biomarker	[19,20]
EBV	Gastric cancer	Candidate immune-sensitive subtype	EBER or validated molecular assay	Exploratory subgroup evidence	Low prevalence and limited prospective validation	[21]
Spatial immune signature	Gastric cancer	Candidate response enrichment	Multiplex IHC and spatial analysis	Single-center retrospective	Platform dependence and no prospective external validation	[64]
Methylation phenotype	BTC/GBC	Prognostic and immune-context association	Research methylome signature	Retrospective integrative study	No ICI-treated validation cohort	[53]
On-treatment vaccine immune response	PDAC and mixed PDAC/CRC vaccine cohorts	Pharmacodynamic association	Study-specific T-cell assays	Early single-arm evidence	Post-treatment definition cannot serve as pretreatment selection test	[58,60]

## 10. Immune-Related Adverse Events and Patient-Centered Considerations

### 10.1. Recognition and Management of Immune-Related Adverse Events

Immune-related adverse events should be treated as a longitudinal management problem rather than as a binary safety label. Baseline assessment should document autoimmune disease, organ function, concomitant medication, and pre-existing symptoms. Surveillance must continue during therapy and after discontinuation because toxicities may be delayed, and some endocrinopathies can be permanent. Combination regimens also require attribution: cytopenias and neuropathy may arise from chemotherapy, bleeding and hypertension from anti-VEGF therapy, and hepatitis, colitis, pneumonitis, myocarditis, or endocrine dysfunction from immune activation [65,66,67].

General management principles are grade and organ specific. Grade 1 toxicities can often be monitored without interruption, except when cardiac, neurologic, hematologic, or selected ocular involvement creates disproportionate risk. Most grade 2 events warrant temporary interruption and consideration of prednisone 0.5–1 mg/kg/day or equivalent. Grade 3 events generally require treatment suspension, urgent specialist input, and prednisone or methylprednisolone 1–2 mg/kg/day, followed by a slow taper of at least four to six weeks once improvement occurs. Steroid-refractory disease requires organ-specific immunosuppression. Grade 4 toxicities usually lead to permanent discontinuation, with controlled endocrinopathies as a common exception. Immune checkpoint inhibitors are interrupted or discontinued rather than dose reduced [65,66].

Absolute toxicity should accompany efficacy. In CheckMate 649, grade 3–4 treatment-related adverse events occurred in 59% of patients receiving nivolumab plus chemotherapy and 44% receiving chemotherapy, with treatment-related death in 2% and 1%, respectively. In KEYNOTE-177, severe adverse events were substantially less frequent with pembrolizumab than with chemotherapy. In contrast, dual-checkpoint strategies can reduce chemotherapy-associated toxicity while introducing colitis, hepatitis, endocrine, and other immune toxicities. The relevant comparison is, therefore, regimen specific, not simply immunotherapy versus no immunotherapy [22,68].

### 10.2. Negative Trials and Treatment Burden

Several randomized failures define the limits of current strategies. Pembrolizumab plus chemotherapy did not meet superiority end points in KEYNOTE-062, atezolizumab with or without cobimetinib failed to improve survival over regorafenib in IMblaze370, dual-checkpoint blockade did not improve survival in unselected PDAC in PA.7, and lenvatinib plus pembrolizumab did not cross the prespecified significance boundaries in LEAP-002. Updated IMbrave050 results further show that an early recurrence-free survival signal can disappear with longer follow-up. These examples justify requiring mature, multiplicity-controlled randomized evidence before early response, pathological response, or translational signals are called practice changing [17,31,39,42,55].

### 10.3. Quality of Life, Cost, and Access

Patient-reported outcomes add information that adverse-event tables cannot provide. In CheckMate 649, nivolumab plus chemotherapy maintained or improved several on-treatment health-related quality-of-life measures, although the analyses were exploratory and vulnerable to open-label and attrition bias. In TOPAZ-1, adding durvalumab did not worsen global health status or quality of life, but it did not produce a statistically clear improvement in time to deterioration; non-detriment should not be described as quality-of-life superiority [69,70].

Cost-effectiveness estimates vary with jurisdiction, drug price, duration, utility assumptions, and willingness-to-pay threshold. Models based on CheckMate 649 and TOPAZ-1 have shown strong sensitivity to checkpoint-inhibitor acquisition cost and should not be generalized globally or indefinitely. Access also depends on biomarker testing, tissue availability, infusion capacity, multidisciplinary toxicity management, and the ability to deliver urgent specialist care. Personalized vaccines and cell therapies add sequencing, bespoke manufacturing, cold-chain, lymphodepletion, hospitalization, and treatment-window constraints. These factors are components of clinical translatability rather than peripheral implementation issues [71,72].

## 11. Artificial Intelligence, Multi-Omics, and Spatial Biology

Artificial intelligence and multi-omics can extend biomarker resolution beyond single analytes, but current studies occupy different levels of readiness. A histology-based deep-learning model for MSI achieved a patient-level area under the curve of 0.81 in a gastric TCGA test set but only 0.69 in an independent Japanese cohort, illustrating domain shift across populations and specimen workflows. Such systems may support prescreening or quality control but cannot replace validated IHC, PCR, or sequencing-based MSI/MMR testing without prospective clinical-utility studies [73].

Spatial profiling can capture immune-cell density and neighborhood relationships that bulk measurements lose. In a single-center study of 80 gastric cancers, including 60 immunotherapy-treated patients, a multiplex-immunohistochemistry signature combining cellular abundance and spatial organization was associated with response and survival. The study demonstrates biological value but remains limited by sample size, retrospective design, platform dependence, and the absence of prospective external validation [64].

Analyses embedded in randomized trials provide stronger clinical context but remain exploratory. In CheckMate 649, only subsets of the randomized population had evaluable whole-exome or RNA sequencing. Hypermutation, MSI, low stromal expression, and other immune programs were associated with differential benefit, but the analyses were post hoc and not multiplicity confirmed. They support prospective biomarker hypotheses rather than new companion diagnostics [21].

Recent multimodal models show both promise and fragility. A model integrating radiology, pathology, and clinical data predicted response to anti-HER2 therapy or anti-HER2 plus immunotherapy in HER2-positive gastric cancer, while an interpretable radiomics model predicted pathological complete response after neoadjuvant immunotherapy in dMMR/MSI-H colorectal cancer. The latter independent validation cohort contained only 22 patients. Before clinical adoption, models require locked algorithms, external multicenter validation, calibration, decision-curve analysis, prospective evaluation, transparent handling of missing data, and testing against existing clinical biomarkers [74,75].

The most credible near-term role for these technologies is layered enrichment: validated clinical assays define the eligible population, while spatial, radiomic, or multi-omic features refine prognosis or trial selection. Replacing standard testing is a higher evidentiary bar that requires demonstration of analytical validity, clinical validity, clinical utility, reproducibility, and equitable performance across sites and populations.

## 12. Cross-Cancer Synthesis and Future Directions

The therapeutic maturity of digestive cancer immunotherapy follows a gradient (Table 8). Advanced esophageal, gastric, HCC, and BTC settings contain phase III standards, although the benefit depends on histology, PD-L1 definition, bleeding risk, liver function, and anatomic site. MSI-H/dMMR colorectal cancer represents the strongest biomarker-defined paradigm and now spans metastatic, neoadjuvant, organ-preservation, and adjuvant settings. By contrast, MSS colorectal cancer and unselected PDAC remain areas in which early immune activation rarely translates into validated survival benefit.

**Table 8:** Cross-cancer comparison of therapeutic maturity, biomarkers, and current challenges.

Cancer and Setting	Established or Leading Strategy	Selection Biomarker	Efficacy Anchor	KEY Toxicity or Burden	Current Challenge	Source
Advanced esophageal cancer	PD-1 inhibitor plus chemotherapy; selected dual ICI	Trial-specific PD-L1 score	KEYNOTE-590 5-year OS 10.6% vs. 3.0%; CheckMate-648 OS benefit	Chemotherapy toxicity or dual-ICI irAEs	Assay harmonization and perioperative survival maturity	[6,7]
Advanced gastric/GEJ cancer	PD-1 inhibitor plus chemotherapy	PD-L1 CPS; MSI-H	CheckMate 649 CPS at least 5 OS HR 0.70	Prolonged combination therapy	Defining benefit at low PD-L1 and integrating HER2/CLDN18.2	[14]
CLDN18.2-positive gastric/GEJ cancer	Zolbetuximab plus chemotherapy	CLDN18.2 IHC	SPOTLIGHT and GLOW PFS/OS benefit	Nausea, vomiting, infusion burden	Sequencing with checkpoint and HER2 strategies	[19,20]
MSI-H/dMMR metastatic CRC	Pembrolizumab or nivolumab plus ipilimumab	MSI-H/dMMR	KEYNOTE-177 PFS HR 0.60; CheckMate 8HW 24-month PFS 72% vs. 14%	Immune toxicity; early progression in a subset	Optimal monotherapy vs. combination selection	[22,24]
Localized dMMR colon cancer	Neoadjuvant ICI; adjuvant atezolizumab plus mFOLFOX6	dMMR	NICHE-2 pCR 68%; ATOMIC 3-year DFS 86.3% vs. 76.2%	Longer treatment and added toxicity in adjuvant therapy	Organ preservation, duration, and long-term survival	[26,30]
MSS/pMMR CRC	No established conventional ICI strategy	No validated marker	Botensilimab phase I ORR 17%	Investigational immune toxicity	Overcoming stromal exclusion and validating liver-metastasis associations	[33]
Unresectable HCC	Atezolizumab–bevacizumab or STRIDE	No validated selection biomarker	IMbrave150 OS HR 0.66; STRIDE 5-year OS 19.6%	Bleeding/vascular risk or dual-ICI irAEs	Liver-function heterogeneity and perioperative failure	[36,38]
Advanced BTC including GBC	Durvalumab or pembrolizumab plus gemcitabine/cisplatin	No validated PD-L1 threshold	TOPAZ-1 OS HR 0.80; KEYNOTE-966 OS HR 0.83	Chemotherapy toxicity and prolonged treatment	Anatomic-subgroup heterogeneity and modest absolute benefit	[45,48]
Unselected PDAC	No established ICI strategy	MSI-H/dMMR for rare exception	PA.7 negative; early vaccine immunogenicity only	Manufacturing, timing, and combination toxicity	Converting immune exclusion into randomized clinical benefit	[55,58]

Future progress requires fewer but more informative trials. Randomized comparisons should use biomarker-enriched populations, mature time-to-event end points, patient-reported outcomes, and prespecified analyses of organ involvement and resistance. Perioperative studies should distinguish pathological response from event-free and overall survival and should measure whether treatment enables safe organ preservation. Early-phase vaccine and cellular studies should report manufacturing failures, treatment-window attrition, dose-limiting toxicities, persistence, and feasibility in addition to immune response.

Biomarker development should move from association to clinical utility. PD-L1, MSI/MMR, HER2, and CLDN18.2 illustrate that assay, threshold, treatment, and disease context must be specified. New spatial, methylation, radiomic, and transcriptional signatures require independent validation and proof that using the test improves a patient-level decision. Similarly, subgroup observations such as the poorer activity of botensilimab in active liver metastasis should motivate prospective stratification, not causal claims.

Finally, therapeutic benefit must be evaluated as a composite of survival, toxicity, function, quality of life, affordability, and access. A regimen that improves a relative hazard while adding prolonged treatment, high-grade toxicity, or specialized manufacturing may still be appropriate, but those costs should be explicit. Precision immunotherapy will mature when evidence hierarchy, biological selection, and patient-centered feasibility are evaluated together.

## Data Availability

The literature search strategy used for the final update is provided in Supplementary Table S1. No new patient-level dataset was generated for this narrative review.

## Supplementary Materials

The following supporting information can be downloaded at: https://www.mdpi.com/article/10.3390/biomedicines14091919/s1, Supplementary Table S1: Structured PubMed Search Strategy and Final Update.

## Abbreviations

AE, adverse event; AI, artificial intelligence; BTC, biliary tract cancer; CAF, cancer-associated fibroblast; CAPOX, capecitabine plus oxaliplatin; CAR-T, chimeric antigen receptor T cell; CI, confidence interval; CPS, combined positive score; CRC, colorectal cancer; CTLA-4, cytotoxic T-lymphocyte-associated protein 4; DFS, disease-free survival; dMMR, deficient mismatch repair; EBV, Epstein–Barr virus; EFS, event-free survival; GBC, gallbladder cancer; GEJ, gastroesophageal junction; HCC, hepatocellular carcinoma; HER2, human epidermal growth factor receptor 2; HR, hazard ratio; HRQoL, health-related quality of life; ICI, immune checkpoint inhibitor; irAE, immune-related adverse event; MHC, major histocompatibility complex; MPR, major pathological response; MSI-H, microsatellite instability-high; MSS, microsatellite stable; ORR, objective response rate; OS, overall survival; PD-1, programmed cell death protein 1; PD-L1, programmed death-ligand 1; PDAC, pancreatic ductal adenocarcinoma; pCR, pathological complete response; PFS, progression-free survival; pMMR, proficient mismatch repair; PRO, patient-reported outcome; QoL, quality of life; RFS, recurrence-free survival; TIL, tumor-infiltrating lymphocyte; TMB, tumor mutational burden; TME, tumor microenvironment.
