# Supplementary material for: Immunotherapy for Digestive System Cancers: Progress, Challenges, and Future Directions
Source: Biomedicines. 2026 Aug 27;14(9):1919. doi: 10.3390/biomedicines14091919 (PMC13604390; doi:10.3390/biomedicines14091919)
Supplement: Supplementary file 1 [file biomedicines-14-01919-s001.zip › biomedicines-4410523-supplementary.pdf]

Supplementary Table S1. Structured PubMed Search Strategy and Final Update

Review framework

The article is a structured narrative review, not a systematic review or meta-analysis. The search was designed to identify pivotal randomized trials, prospective clinical studies, mature follow-up reports, clinically relevant negative studies, original translational studies, patient-reported outcome studies, cost-effectiveness analyses, and authoritative toxicity-management guidelines. Reference lists of pivotal trials and relevant guidelines were also checked to identify primary reports and mature updates. No claim of exhaustive study identification is made.

Database and final update

- Database: PubMed
- Final search date: 4 August 2026
- Supplemental-update publication-year filter: 2025–2026
- Language used for the searches: English
- Number of targeted queries: 10
- Unique records returned after cross-query deduplication: 68

Exact targeted queries used for the final update

No.	Exact PubMed query	Records retained after the 2025–2026 post-filter
1	esophageal cancer immunotherapy phase 3 clinical trial	8
2	gastric gastroesophageal cancer immunotherapy phase 3 clinical trial	8
3	colorectal cancer immunotherapy phase 2 phase 3 clinical trial	8
4	hepatocellular carcinoma immunotherapy phase 3 clinical trial	7
5	biliary tract gallbladder cancer immunotherapy phase 2 phase 3 clinical trial	4
6	pancreatic ductal adenocarcinoma immunotherapy vaccine phase 1 phase 2 clinical trial	4
7	gastrointestinal cancer immune-related adverse events management	5
8	gastrointestinal cancer artificial intelligence immunotherapy response multiomics spatial	16
9	gastrointestinal cancer immunotherapy quality of life cost effectiveness	5
10	digestive cancer immunotherapy negative trial resistance	8

The per-query counts are not additive because the same record could be retrieved by more than one query. After cross-query deduplication, 68 unique records remained for title/abstract assessment and citation checking.

Evidence-selection principles

Priority was given to peer-reviewed randomized trials, prospective clinical studies, mature follow-up reports, original translational analyses, patient-reported outcome studies, health-economic analyses, and current consensus guidelines. Meeting abstracts were retained only when no peer-reviewed full report was available and were labelled as such. Case reports and very small case series were not used to support treatment recommendations. Preclinical studies were used only to explain mechanisms or strategies that remain preclinical. Evidence was synthesized narratively; no pooled effect estimate or formal risk-of-bias instrument was used.
